# A Combination of Mitochondrial Oxidative Stress and Excess Fat/Calorie Intake Accelerates Steatohepatitis by Enhancing Hepatic CC Chemokine Production in Mice

**DOI:** 10.1371/journal.pone.0146592

**Published:** 2016-01-08

**Authors:** Tadashi Moro, Sachie Nakao, Hideaki Sumiyoshi, Takamasa Ishii, Masaki Miyazawa, Naoaki Ishii, Tadayuki Sato, Yumi Iida, Yoshinori Okada, Masayuki Tanaka, Hideki Hayashi, Satoshi Ueha, Kouji Matsushima, Yutaka Inagaki

**Affiliations:** 1 Center for Matrix Biology and Medicine, Graduate School of Medicine, Tokai University, Isehara, Kanagawa, Japan; 2 Department of Regenerative Medicine, Tokai University School of Medicine, Isehara, Kanagawa, Japan; 3 Department of Molecular Life Science, Tokai University School of Medicine, Isehara, Kanagawa, Japan; 4 Support Center for Medical Research and Education, Research and Promotion Division (Isehara), Tokai University, Isehara, Kanagawa, Japan; 5 Institute of Medical Sciences, Tokai University, Isehara, Kanagawa, Japan; 6 Research Laboratory, Minophagen Pharmaceutical Co. Ltd., Zama, Kanagawa, Japan; 7 Department of Molecular Preventive Medicine, Graduate School of Medicine, University of Tokyo, Bunkyo-ku, Tokyo, Japan; 8 CREST, Japan Science Technology/Japan Agency for Medical Research and Development, Chiyoda-ku, Tokyo, Japan; Institute of Medical Research A Lanari-IDIM, University of Buenos Aires-National Council of Scientific and Technological Research (CONICET), ARGENTINA

## Abstract

Mitochondrial oxidative stress is considered as a key accelerator of fibrosis in various organs including the liver. However, the production of oxidative stress and progression of liver fibrosis may merely represent the independent consequences of hepatocellular injury caused by the primary disease. Because of a lack of appropriate experimental models to evaluate the sole effects of oxidative stress, it is virtually unknown whether this stress is causatively linked to the progression of liver fibrosis. Here, we examined the direct effects of mitochondrial reactive oxygen species (ROS) on the progression of high fat/calorie diet-induced steatohepatitis using *Tet-mev-1* mice, in which a mutated succinate dehydrogenase transgene impairs the mitochondrial electron transport and generates an excess amount of ROS in response to doxycycline administration. Wild type and *Tet-mev-1* mice that had been continuously given doxycycline-containing water were subsequently fed either normal chow or a cholesterol-free high-fat/high-sucrose diet for 4 months at approximately 1 or 2 years of age. Histopathological examinations indicated that neither the mitochondrial ROS induced in *Tet-mev-1* mice nor the feeding of wild type animals with high-fat/high-sucrose diet alone caused significant liver fibrosis. Only when the *Tet-mev-1* mice were fed a high-fat/high-sucrose diet, it induced lipid peroxidation in hepatocytes and enhanced hepatic CC chemokine expression. These events were accompanied by increased infiltration of CCR5-positive cells and activation of myofibroblasts, resulting in extensive liver fibrosis. Interestingly, this combinatorial effect of mitochondrial ROS and excess fat/calorie intake on liver fibrosis was observed only in 2-year-old *Tet-mev-1* mice, not in the 1-year-old animals. Collectively, these results indicate that mitochondrial ROS in combination with excess fat/calorie intake accelerates liver fibrosis by enhancing CC chemokine production in aged animals. We have provided a good experimental model to explore how high fat/calorie intake increases the susceptibility to nonalcoholic steatohepatitis in aged individuals who have impaired mitochondrial adaptation.

## Introduction

Mitochondria are the major source of reactive oxygen species (ROS), where ROS are generated as a byproduct of the electron transport chain during aerobic metabolism. An excess amount of ROS is usually balanced by the presence of a scavenging system including several antioxidants and antioxidant enzymes such as superoxide dismutase and glutathione peroxidase [1]. However, oxidative stress caused by a disruption of this equilibrium induces damage and senescence of normal cells, leading to a number of pathophysiological conditions such as aging, inflammatory diseases, diabetes, and cancer [2, 3].

Oxidative stress has also been implicated in the progression of various liver diseases including nonalcoholic steatohepatitis (NASH) [4], alcoholic liver disease [1], and chronic hepatitis C [5]. In particular, the prevalence of NASH is rapidly increasing in both developed and developing countries and has become a serious health issue worldwide [6]. NASH develops in certain populations (10–20%) of patients with nonalcoholic fatty liver disease, which is characterized by inflammatory cell infiltration, progressive fibrosis, and an increased risk of hepatocellular carcinoma [7]. However, the subgroups of patients with nonalcoholic fatty liver disease that go on to develop NASH and the key factors that accelerate the disease progression have not yet been clearly identified. A “two-hit” theory has been proposed as the underlying mechanism responsible for NASH development; the first hit is excessive fat/calorie intake leading to simple steatosis, and the second hit includes oxidative stress caused by mitochondrial dysfunction [8]. Indeed, mitochondrial abnormalities have been implicated in the development of NASH [9, 10], and several studies have indicated a strong association between the degree of oxidative stress and the disease severity of NASH [11, 12].

However, the causal relationship between oxidative stress and hepatic fibrogenesis remains poorly understood because the production of oxidative stress and the progression of liver fibrosis may merely represent independent consequences of hepatocellular injury caused by the primary disease. Previous studies have used several experimental models such as carbon tetrachloride intoxication and a methionine-choline deficient diet, both of which not only induce a large amount of oxidative stress but also cause extensive hepatocellular necrosis/apoptosis. Because of a lack of appropriate experimental models to evaluate the sole effects of oxidative stress, it is virtually unknown whether this stress is linked directly and causatively to the progression of liver fibrosis.

In the present study, we aimed to examine whether mitochondrial oxidative stress *per see* contributes to hepatic fibrogenesis under a physiological condition without significant hepatocellular injury, and to explore how high fat/calorie intake, which does not induce significant fibrosis by itself, affects the progression of steatohepatitis. For this purpose, we used a transgenic mouse strain (*Tet-mev-1*) that harbors a mutated transgene coding for the mitochondrial succinate dehydrogenase cytochrome *b* large subunit (SDHC) under the control of a unique Tet-on/off system [13]. This mutant triggers a leakage of an electron from the mitochondrial respiratory chain, and a superoxide anion is generated from the leaked electron and oxygen.

Our results indicated that mitochondrial ROS induced by the impaired electron transport *per se* did not cause significant liver injury or fibrosis. However, feeding of *Tet-mev-1* mice with a cholesterol-free high-fat/high-sucrose (HFHS) diet, which did not induce significant fibrosis when given to wild type animals, caused extensive liver fibrosis by increasing lipid peroxidation in hepatocytes and CC chemokine expression in liver tissues. In addition, the progression from simple steatosis to fibrosis was apparent in aged *Tet-mev-1* mice, but not in younger animals, possibly depending on the duration and extent of ROS-induced chemokine production as well as the impaired mitochondrial adaptation in aged mice.

## Materials and Methods

### Transgenic mice and induction of mitochondrial ROS

**Fig 1:**
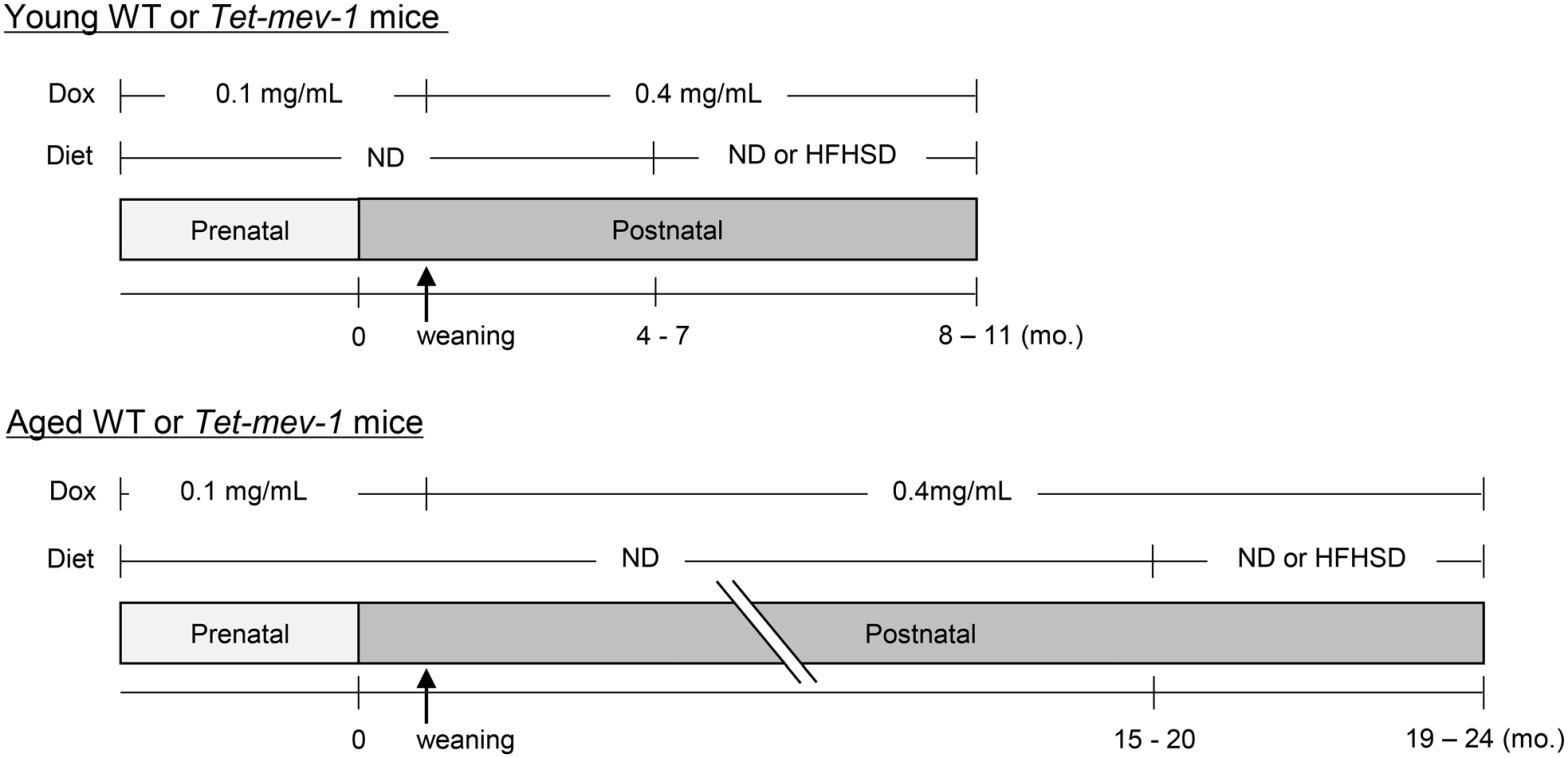
Experimental design. Male wild type (*WT*) and *Tet-mev-1* mice had been continuously supplied with drinking water containing doxycycline *(Dox*) at a concentration of either 0.1 mg/mL during the prenatal period (received through their mothers) or 0.4 mg/mL after weaning. They were divided into four groups (n = 8 in each group) which were subsequently fed a control, normal diet *(ND*) or a high-fat/high-sucrose diet (*HFHSD*) at approximately either 1 (*upper*) or 2 years of age (*lower*). The mice were sacrificed under isoflurane anesthesia at the end of the 4-month feeding period, and the obtained serum and liver specimens were subjected to further analyses.

*Tet-mev-1* transgenic mice on a C57BL/6 background were generated as previously described [13]. All animals used in the present study received humane care, and the experiments were approved by the Animal Experiment Committee of Tokai University (No. 151020). Male wild type and *Tet-mev-1* mice were continuously supplied with drinking water containing doxycycline (Sigma-Aldrich, St. Louis, MO) at a concentration of either 0.1 mg/mL during the embryonic period (through their mother) or 0.4 mg/mL after weaning (Fig 1). By drinking this concentration of doxycycline, up to 50 mg/kg/day of the antibiotic was ingested by the mice, which may in itself cause mitonuclear protein imbalance and mitochondrial dysfunction [14]. Therefore, all experiments were performed with careful consideration to include wild type littermates treated in exactly the same way as *Tet-mev-1* mice. In some experiments, mice were fed a HFHS diet (F2HFHSD, Oriental Yeast, Tokyo, Japan) containing 30% fat, 20% sucrose, and little cholesterol (S1 Table), for 4 months, at approximately either 1 or 2 years of age (Fig 1). A pilot study using wild type and *Tet-mev-1* mice indicated that consumption of a HFHS diet for 4 months caused an approximately 1.5-fold increase in body weight in both young and aged mice, and there was no difference in the body weight increase between wild type and *Tet-mev-1* mice (S2 Table). No mice died prior to the experimental end point. Serum levels of alanine aminotransferase, cholesterol and triglyceride were measured using an automated chemical analyzer, SPOTCHEM EZ (Arkray, Kyoto, Japan).

### Isolation and primary culture of murine hepatocytes

Hepatocytes were isolated from wild type or *Tet-mev-1* mice using the collagenase perfusion method as previously described [15]. The cells were subjected to primary culture using Dulbecco’s modified Eagle medium supplemented with 10% fetal bovine serum. To evaluate the cytotoxicity of doxycycline, primary hepatocytes obtained from wild type and *Tet-mev-1* mice were seeded on a type I collagen-coated 96-well plate (AGC Techno Glass, Shizuoka, Japan) at a density of 5 x 10^4^ cells/cm^2^. The cells were stimulated with various concentrations of doxycycline for 72 hours. Cell viability was estimated using Cell counting kit-8 (DOJINDO Laboratories, Kumamoto, Japan) according to the manufacturer’s instruction.

### Measurement of ROS production and membrane potential of mitochondria

Primary hepatocytes obtained from wild type or *Tet-mev-1* mice that had been supplied with doxycycline-free water were treated *in vitro* with different concentrations of doxycycline for 72 hours immediately after seeding. To estimate mitochondrial ROS production, the cells were incubated with 100 μM dichlorofluorescein diacetate (Sigma-Aldrich) for 30 min., and fluorescence intensities at 520 nm were determined using the GloMax Multi Detection System (Promega, Madison, WI). To detect decreases in the mitochondrial membrane potential, primary hepatocytes were incubated with 300 nM tetramethyl rhodamine methyl ester (Sigma-Aldrich) for 30 min. The fluorescence of tetramethyl rhodamine methyl ester at 573 nm was imaged using a confocal laser scanning microscope LSM510meta (Carl Zeiss, Jena, Germany) and was semi-quantified by measuring the fluorescence intensity.

### Histological and immunohistological studies

Serial sections prepared from the excised liver were subjected to hematoxylin and eosin staining and Sirius red-Fast green FCF staining using standard protocols. An authorized hepatologist (YI) examined the liver tissues in a blinded manner and estimated the presence and degree of inflammation and fibrosis primarily based on the NASH Clinical Research Network Scoring System that had been established for human NASH [16]. The degree of fibrosis in the hepatic parenchyma was also semi-quantified by measuring the mean relative areas stained positive for Sirius red with the aid of ImageJ software (National Institutes of Health, Bethesda, MD) as previously described [17]. For the immunohistological analyses, liver sections were subjected to optimal antigen retrieval using citric acid buffer (pH 6.0) for 10 min at 98°C. After blocking the endogenous peroxidase activity and non-specific protein binding, the sections were incubated for 30 min with specific primary antibodies for 4-hydroxy-2-nonenal (4-HNE)-modified proteins (Japan Institute for the Control of Aging, Shizuoka, Japan), mouse CCR5 (Santa Cruz Biotechnology, Santa Cruz, CA) or mouse α-smooth muscle actin (αSMA; Sigma-Aldrich). After incubation with horseradish-conjugated secondary antibodies, they were visualized using diaminobenzidine (Dako, Glostrup, Denmark) with subsequent hematoxylin counter-staining. Apoptotic cells were detected by the TdT-mediated dUTP nick end labeling method using TACS2 TdT-DAB *In Situ* Apoptosis Detection Kit (Trevigen, Gaithersburg, MD).

### Fluorescence activated cell sorting analyses

Whole blood was collected from mice through the inferior vena cava, and the peripheral blood mononuclear cells were isolated by density centrifugation using Histopaque-1083 (Sigma-Aldrich). The cells were stained with an antibody cocktail consisting of APC/Cy7 anti-mouse F4/80 (BioLegend, San Diego, CA), PE/Cy7 anti-mouse/human CD11b (BioLegend), PE anti-mouse Ly-6C (BD Pharmingen, Franklin Lakes, NJ), and APC anti-mouse CD195/CCR5 (BioLegend). Activated profibrogenic monocytes/macrophages were identified as an F4/80^+^/CD11b^+^/Ly-6C^high^ cell fraction [18] and collected using BD FACS Aria cell sorter (Becton Dickinson, San Jose, CA). CCR5 expression in these cells was analyzed using FlowJo software (TOMY Digital Biology, Tokyo, Japan).

### Expression microarray and real time reverse-transcription polymerase chain reaction (RT-PCR) analyses

Total RNA was extracted from liver tissues using RNeasy Mini kit (Qiagen, Valencia, CA). Extracted RNA obtained from wild type and *Tet-mev-1* mice (n = 2 per group) was labeled and hybridized to the Whole Mouse Genome Microarray Kit (Agilent Technology, Santa Clara, CA). The obtained microarray data have been submitted to Gene Expression Omnibus (ID: GSE68632). Functional annotation of gene ontology was performed using GeneSpring GX software (Agilent Technology). To quantify the amounts of mRNA, the total RNA isolated from the liver tissues was reverse-transcribed and amplified as previously described [17] using SYBR Green PCR master mix (Applied Biosystems, Foster City, CA) and the specific primers shown in S3 Table. The gene expression levels in each sample were normalized against those of glyceraldehyde 3-phosphate dehydrogenase mRNA.

### Statistical analyses

Values were expressed as mean ± SD. Statistical analyses were performed using the Kruskal-Wallis’ test, and the Mann-Whitney U test was used in a post-test comparison to evaluate the differences between the groups. A *p* value less than 0.05 was considered statistically significant.

## Results

### Cytotoxicity of doxycycline to primary cultures of hepatocytes

**Fig 2:**
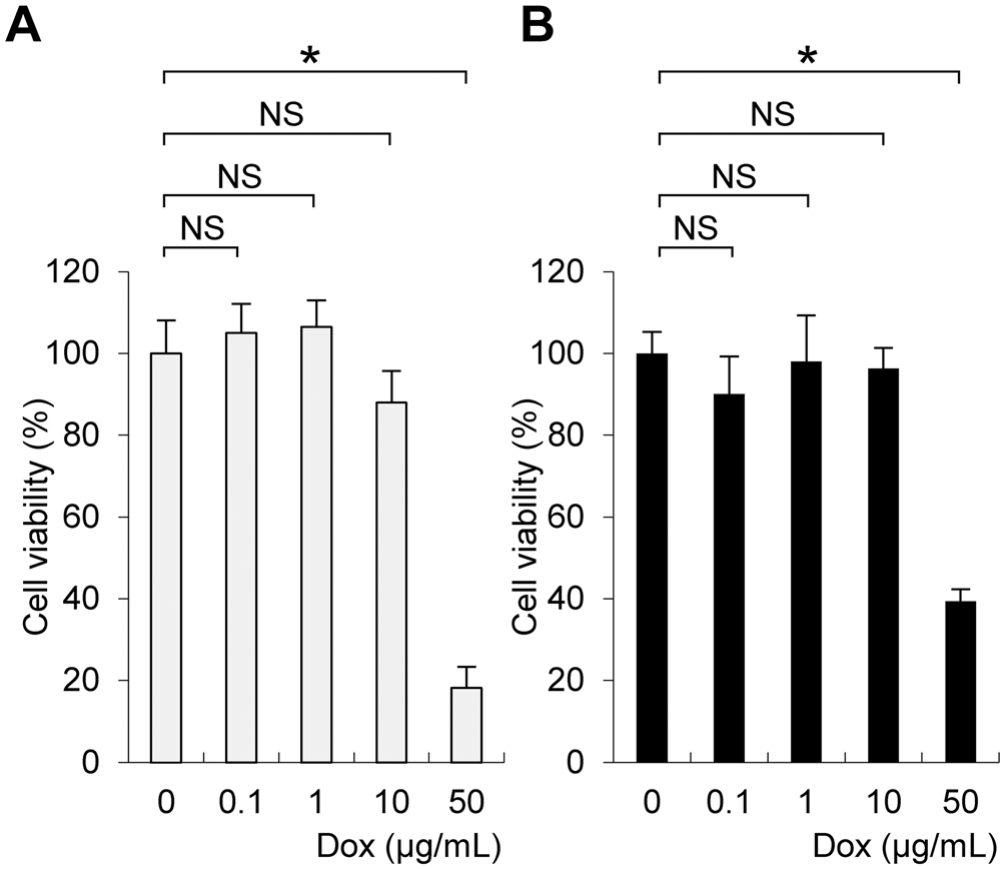
Cytotoxicity of doxycycline to primary cultures of hepatocytes. Hepatocytes were isolated from 9-week-old female wild type (**A**) or *Tet-mev-1* mice (**B**) that had been supplied with doxycycline-free water. The cells were subjected to primary culture, treated with the indicated concentrations of doxycycline (*Dox*) for 72 hours, and then examined using cell counting assays. The values are expressed as means ± SD from six samples in each group. The asterisk indicates that the difference is statistically significant between the groups (*p < 0.01). NS, not significant.

We first determined the cytotoxicity of doxycycline to primary murine hepatocytes. For this purpose, the viability of primary hepatocytes isolated from wild type and *Tet-mev-1* mice was examined after treatment with various concentrations of doxycycline. The results indicated that concentrations of doxycycline up to 10 μg/mL did not affect the survival of the hepatocytes obtained from either the wild type (Fig 2A) or *Tet-mev-1* mice (Fig 2B). Based on these findings, doxycycline concentrations within the non-cell-killing range were used to treat primary hepatocytes in further *in vitro* experiments.

### ROS induction and decreased mitochondrial membrane potential were observed in *Tet-mev-1* hepatocytes

**Fig 3:**
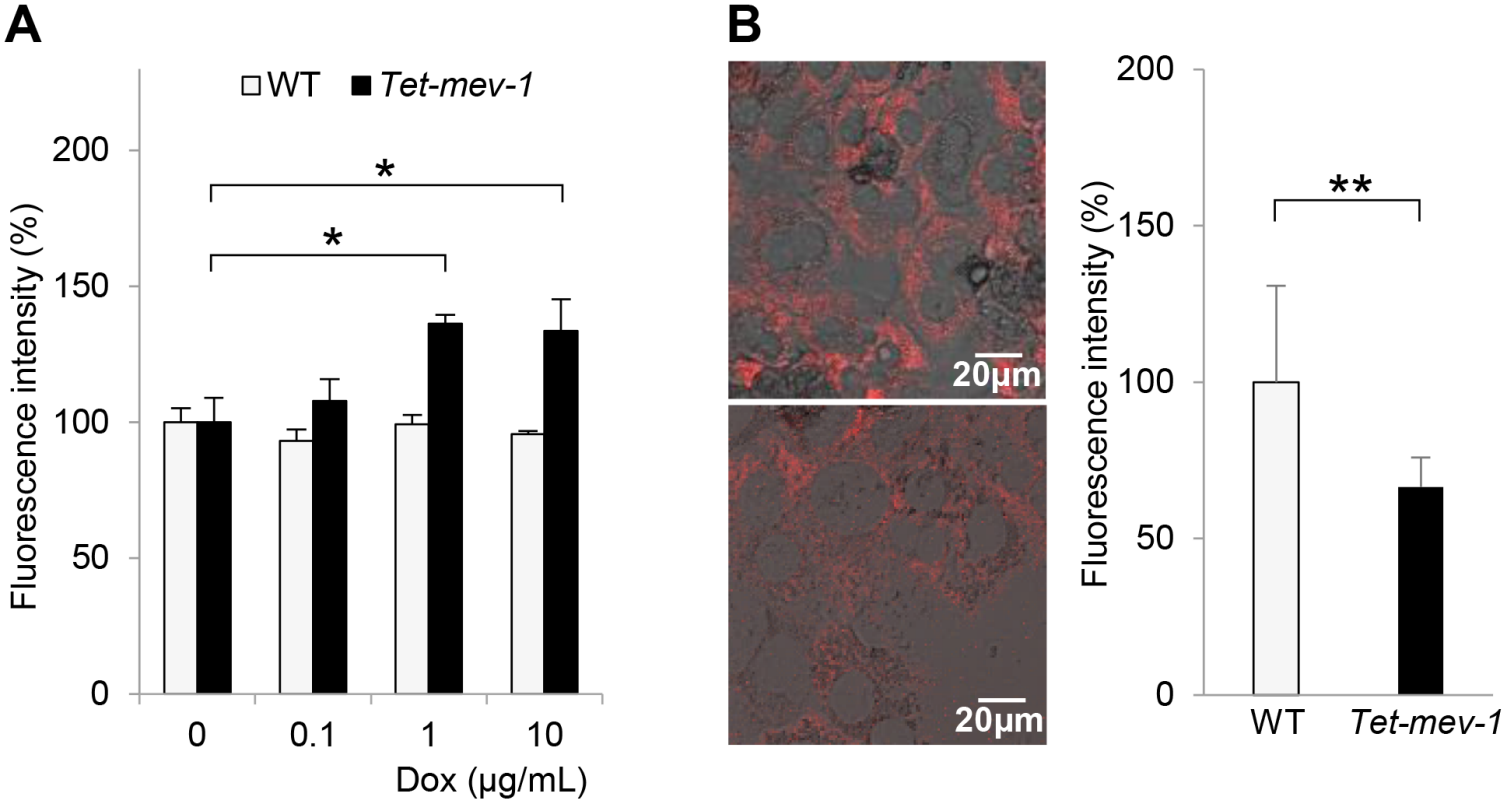
Increased reactive oxygen species (ROS) production and decreased mitochondrial membrane potential in *Tet-mev-1* hepatocytes. (**A**) Primary hepatocytes were isolated from 14-month-old male wild type (*WT*) or *Tet-mev-1* mice that had been supplied with doxycycline-free water. After treatment with the indicated concentrations of doxycycline (*Dox*) for 72 hours, they were incubated with 100 μM dichlorofluorescein diacetate for 30 min. The fluorescence intensity was measured at 520 nm, and the values are expressed as means ± SD from six samples in each group. (**B**) Hepatocytes that had been isolated from the same mice used for the experiment shown in Fig 3A were incubated with 300 nM tetramethyl rhodamine methyl ester for 30 min. The fluorescence at 573 nm was viewed and analyzed using a confocal laser-scanning microscope with the same excitation strength and detection gain. Representative images are shown for hepatocytes obtained from wild type (*upper*) and *Tet-mev-1* mice (*lower*). *Scale bars*, 20 μm. On the right side of the images, histograms indicating the values (means ± SD) of fluorescence intensities obtained from five randomly selected visual fields in each group are shown. The asterisks indicate that the differences between the groups are statistically significant (*p < 0.01; **p < 0.05).

We then confirmed ROS induction and subsequent mitochondrial dysfunction in the *Tet-mev-1* hepatocytes by doxycycline treatment *in vitro*. Incubation with 1–10 μg/mL of doxycycline increased mitochondrial ROS production by 1.4-fold in hepatocytes derived from the *Tet-mev-1* mice that had been supplied with doxycycline-free water (Fig 3A). In contrast, there was no increase in the ROS production in the wild type hepatocytes that were treated with the same concentrations of doxycycline *in vitro* (Fig 3A). In parallel to the increased ROS production, treatment of the *Tet-mev-1* hepatocytes with 10 μg/mL of doxycycline significantly decreased the mitochondrial membrane potential estimated by tetramethyl rhodamine methyl ester fluorescence from that estimated for the wild type hepatocytes that were treated with the same concentration of doxycycline (Fig 3B).

### Mitochondrial oxidative stress *per se* did not cause liver injury or fibrosis in *Tet-mev-1* mice

**Fig 4:**
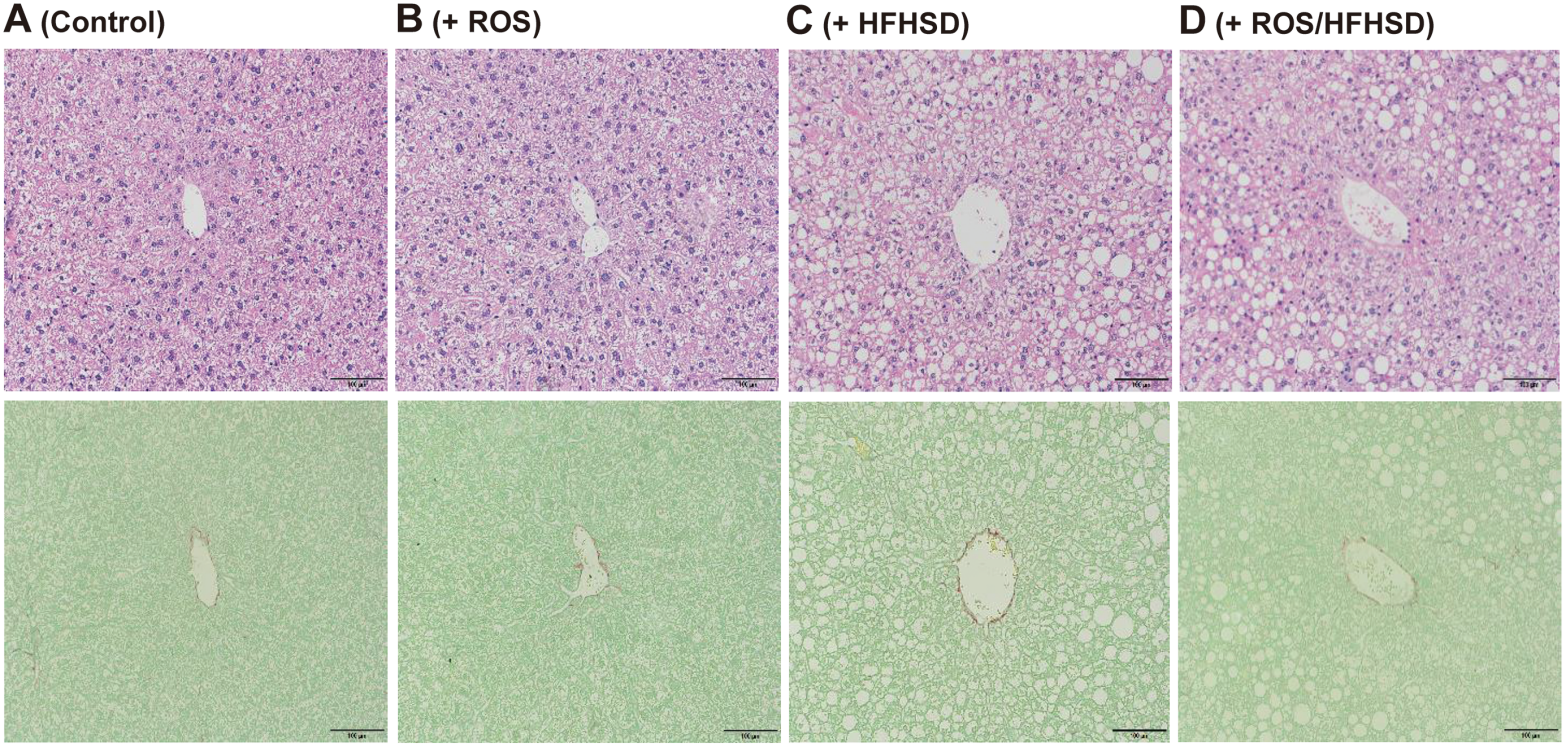
Histopathological findings of liver tissues from young mice fed normal chow or high-fat/high-sucrose diet. Liver specimens were obtained from male wild type (**A** and **C**) or *Tet-mev-1* mice (**B** and **D**) that had been supplied with doxycycline-containing water throughout the prenatal and postnatal periods and, at around the age of 1 year, were subsequently fed a control, normal diet (**A** and **B**) or high-fat/high-sucrose diet (**C** and **D**) for 4 months. Serial sections were subjected to hematoxylin and eosin staining (*upper*) or Sirius red-Fast green FCF staining (*lower*). Representative images from eight mice in each group are shown. *Scale bars*, 100 μm.

**Fig 5:**
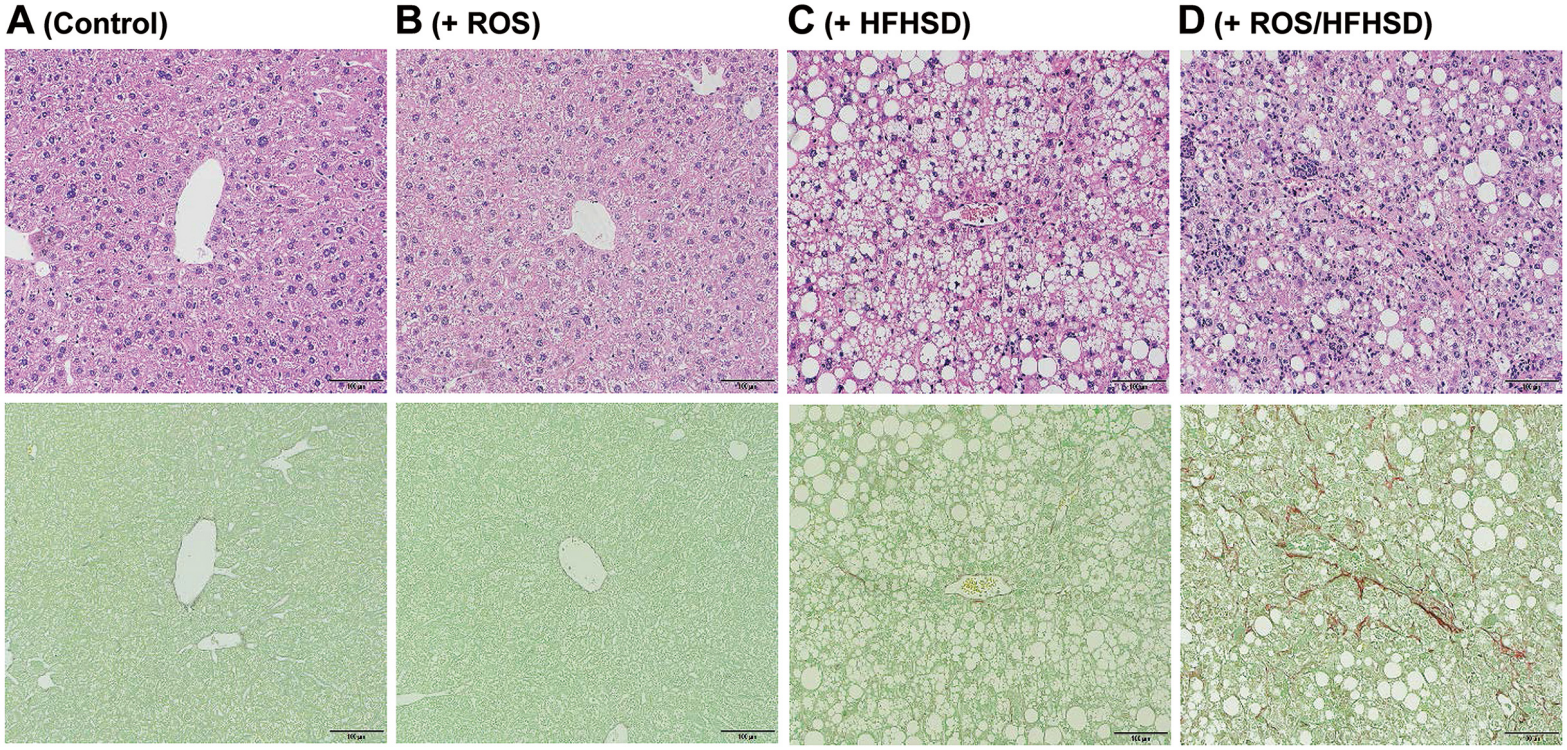
Histopathological findings of liver tissues from aged mice fed normal chow or high-fat/high-sucrose diet. Liver specimens were obtained from male wild type (**A** and **C**) or *Tet-mev-1* mice (**B** and **D**) that had been supplied with doxycycline-containing water throughout the prenatal and postnatal periods and, at around the age of 2 years, were subsequently fed a control, normal diet (**A** and **B**) or high-fat/high-sucrose diet (**C** and **D**) for 4 months. Serial sections were subjected to hematoxylin and eosin staining (*upper*) or Sirius red-Fast green FCF staining (*lower*). Representative images from eight mice in each group are shown. *Scale bars*, 100 μm.

The mitochondrial dysfunction observed in the above *in vitro* experiments with isolated *Tet-mev-1* hepatocytes may not be applicable to the chronic fibrogenic process *in vivo*. We therefore examined whether mitochondrial oxidative stress induced in the *Tet-mev-1* mice affected the serum biochemical parameters and histopathological findings of the liver *in vivo*. Irrespective of the mouse age, there were no significant differences in the mean levels of serum alanine aminotransferase, cholesterol, or triglyceride between the wild type and *Tet-mev-1* mice that had been fed a normal diet (S1 Fig). Consistent with these biochemical data, histological examination of liver specimens obtained from 1- (Fig 4B) and 2-year-old *Tet-mev-1* mice (Fig 5B) that had been given doxycycline-containing water and fed normal chow showed no apparent hepatocellular necrosis or fibrosis when compared with that in their wild type littermates of the same age (Figs 4A and 5A, respectively). These findings indicated that mitochondrial ROS induced in *Tet-mev-1* mice *per se* did not cause significant hepatocellular injury or fibrosis.

### A combination of mitochondrial ROS production and HFHS diet contributed to development of liver fibrosis in aged *Tet-mev-1* mice

**Fig 6:**
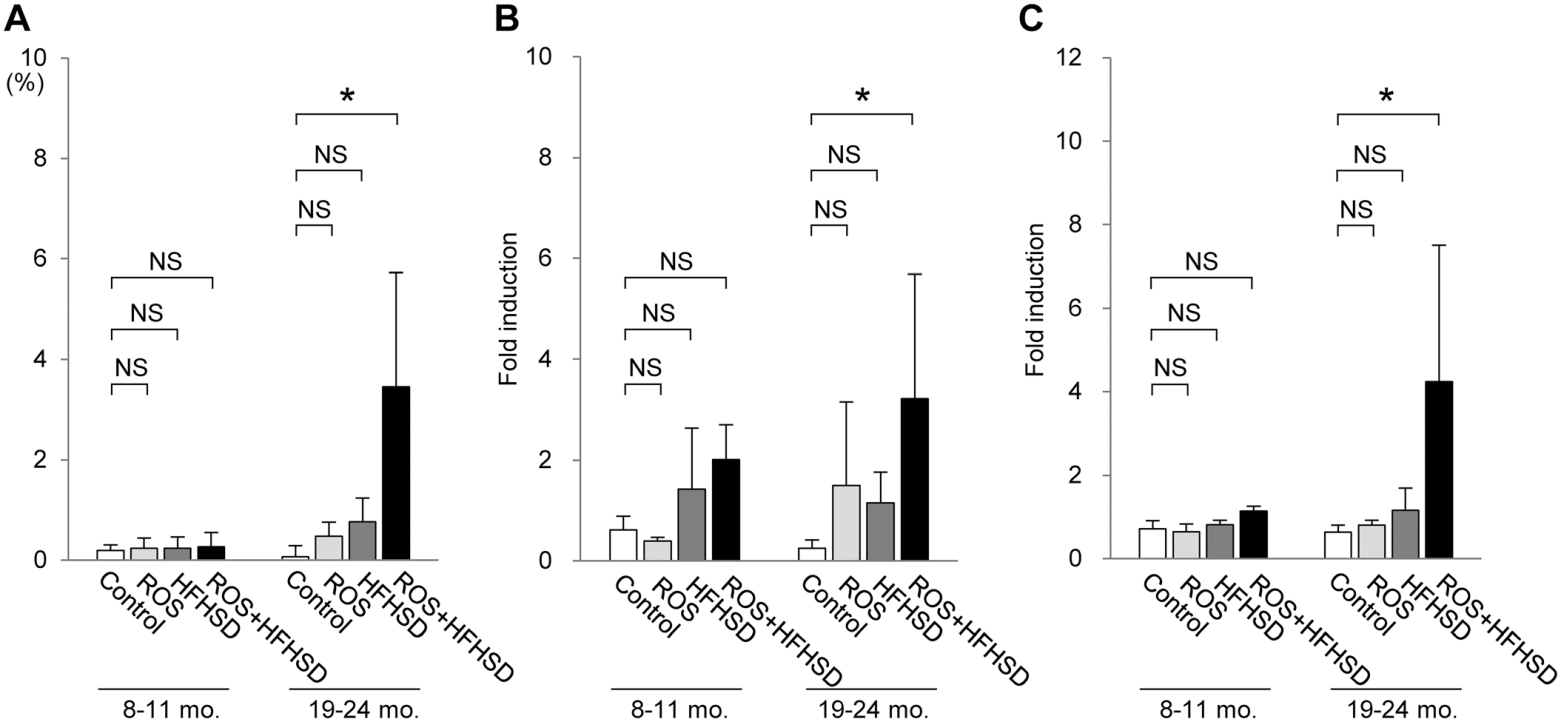
Degree of fibrosis and expression of profibrogenic genes in liver tissues from mice fed normal chow or high-fat/high-sucrose diet. Liver specimens were obtained and the total RNA was extracted from male wild type or *Tet-mev-1* mice (*+ROS*) that had been supplied with doxycycline-containing water and were subsequently fed a control, normal diet or high-fat/high-sucrose diet (+*HFHSD*) for 4 months at around the age of either 1 (*left*) or 2 years (*right*). The degree of liver fibrosis was semi-quantified by measuring the mean relative areas stained positive for Sirius red (**A**), and the gene expression levels of proα1 type I collagen (**B**) and transforming growth factor-β1 (**C**) were quantified using specific primers. The values represent the mean ± SD obtained from eight mice per group. The asterisks indicate that the differences between the groups are statistically significant (*p < 0.05). NS, not significant.

We next tried to explore the effects of excess fat/calorie intake on the progression of steatohepatitis. A similar degree of fatty metamorphosis was observed in the liver of the 1-year-old wild type (upper panel in Fig 4C) and *Tet-mev-1* mice (upper panel in Fig 4D) that had been supplied with doxycycline-containing water and subsequently fed a HFHS diet for 4 months. However, no apparent fibrosis developed in these 1-year-old mice given doxycycline-containing water and a HFHS diet (lower panels in Fig 4C and 4D). In addition, only minimal fibrosis was observed in the liver parenchyma of the 2-year-old wild type mice that had been supplied with doxycycline-containing water and subsequently fed a HFHS diet (lower panel in Fig 5C). In marked contrast to those results, feeding of the 2-year-old *Tet-mev-1* mice, which had been given doxycycline-containing water, with a HFHS diet remarkably increased the number of infiltrating cells in the liver (upper panel in Fig 5D) and accelerated liver fibrosis (lower panel in Fig 5D). Semi-quantitative analyses of the relative fibrotic areas in hepatic parenchyma confirmed that, among the four groups of 2-year-old mice, the degree of liver fibrosis was significantly increased only in the *Tet-mev-1* mice fed a HFHS diet (Fig 6A). In parallel, the expression levels of profibrogenic genes such as type I collagen (Fig 6B) and transforming growth factor-β1 (Fig 6C) were significantly upregulated in the liver tissues of the 2-year-old *Tet-mev-1* mice supplied with doxycycline-containing water and fed a HFHS diet. These results clearly indicated that neither excessive mitochondrial ROS nor high fat/calorie intake alone is sufficient, but a combination of both is required to cause significant liver fibrosis in aged mice.

### Hepatocytes with fatty degeneration were the primary source of mitochondrial ROS in aged *Tet-mev-1* mice

**Fig 7:**
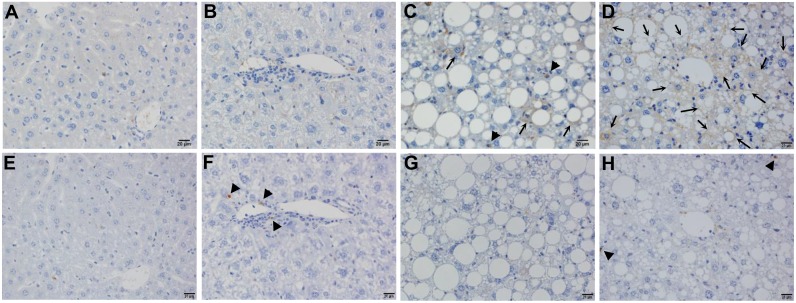
Detection of lipid peroxidation and apoptosis in liver tissues. Liver specimens were obtained from male wild type (**A, C, E,** and **G**) or *Tet-mev-1* mice (**B, D, F** and **H**) that had been supplied with doxycycline-containing water and were subsequently fed a control, normal diet (**A, B, E,** and **F**) or high-fat/high-sucrose diet (**C, D, G** and **H**) for 4 months at around the age of 2 years. They were subjected to 4-hydroxy-2-nonenal staining (**A** to **D**) or apoptosis detection using the TdT-mediated dUTP nick end labeling method (**E** to **H**). Hepatocytes and non-parenchymal cells positively stained for 4-hydroxy-2-nonenal are indicated by arrows and arrowheads, respectively, in panels **C** and **D**, while non-parenchymal cells showing apoptosis are shown by arrowheads in panels **F** and **H**. Representative images from five mice in each group are shown. *Scale bars*, 20 μm.

Immunohistochemical staining of 4-HNE showed no lipid peroxidation in the liver tissues from the 2-year-old wild type (Fig 7A) and *Tet-mev-1* mice (Fig 7B) fed a normal diet. Only a small number of hepatocytes and non-parenchymal cells were positively stained for 4-HNE in the wild type animals that had been fed a HFHS diet (Fig 7C). In contrast, remarkable lipid peroxidation was detected in the parenchymal hepatocytes with fatty degeneration in the 2-year-old *Tet-mev-1* mice fed a HFHS diet (Fig 7D). These results therefore indicated that parenchymal hepatocytes are the main cellular target of mitochondrial stress in aged *Tet-mev-1* mice fed a HFHS diet. On the other hand, no apoptosis was observed in the liver tissues from 2-year-old wild type mice that had been fed a normal diet (Fig 7E) or HFHS diet (Fig 7G), and only a small number of non-parenchymal cells, but not parenchymal hepatocytes, showed apoptosis in the *Tet-mev-1* mice, irrespective of the type of diet provided (Fig 7F and 7H).

### Mitochondrial oxidative stress in combination with excess fat/calorie intake stimulated CC chemokine expression in liver tissues

**Fig 8:**
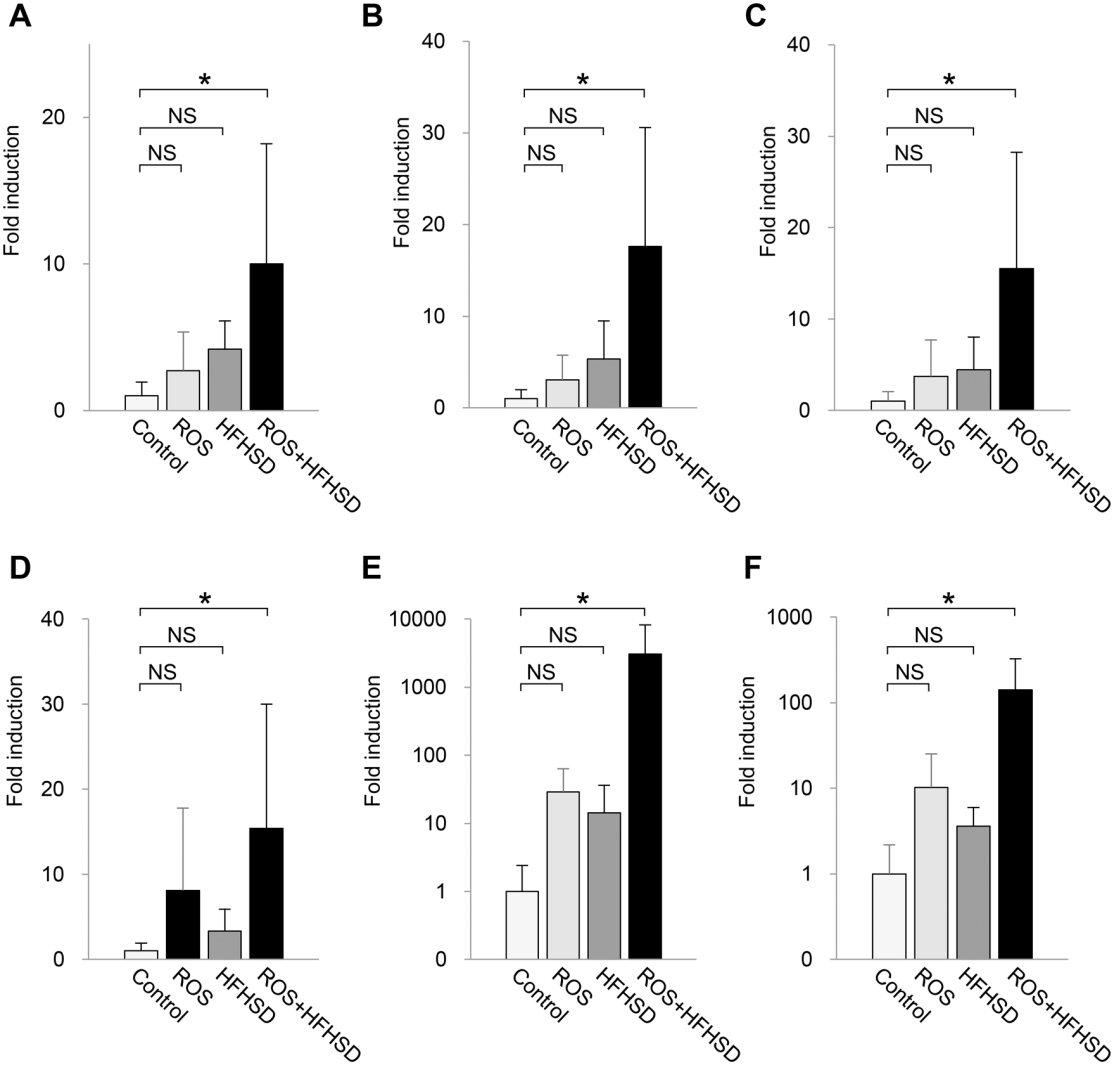
CC chemokine expression in liver tissues from mice fed normal chow or high-fat/high-sucrose diet. Total RNA was extracted from liver tissues of male wild type or *Tet-mev-1* mice (*+ROS*) that had been supplied with doxycycline-containing water and were subsequently fed a control, normal diet or high-fat/high-sucrose diet (*+HFHSD*) for 4 months at around the age of either 1 (*left*) or 2 years (*right*). The gene expression levels of CCL2 (**A**), CCL3 (**B**), CCL4 (**C**), CCL5 (**D**), CCL8 (**E**) and CCL12 (**F**) were quantified using specific primers. The values are expressed as means ± SD from four to five mice per group. The asterisks indicate that the differences between the groups are statistically significant (*p < 0.05). NS, not significant.

In the next set of experiments, we examined how mitochondrial ROS induced in *Tet-mev-1* mice affected the gene expression profile in the liver and accelerated fibrosis when the mice were fed a HFHS diet. For this purpose, total RNA was extracted from the liver tissues of the 1-year-old wild type and *Tet-mev-1* mice that had been given doxycycline-containing water. Microarray analyses showed no difference in the profiles between the wild type and *Tet-mev-1* mice fed a normal diet, indicating that the excessive ROS production induced by the mutated SDHC *per se* did not cause any significant change in the gene expression profile in the liver (Table 1). Furthermore, the wild type and *Tet-mev-1* mice fed a HFHS diet did not show any significant differences in the hepatic gene expression profile. On the other hand, when the wild type mice were fed a HFHS diet, the expression of 69 genes changed more than 10-fold from that in the mice fed normal chow and, as expected, 11 of these genes were related to the lipid metabolic process (Table 1). More importantly, in the *Tet-mev-1* mice fed a HFHS diet, the expression of 208 genes showed changes of over 5-fold as compared with the same transgenic animals fed a normal diet. Gene ontology analyses indicated significant changes in chemokine activity and chemokine receptor binding as well as defense and immune responses (Table 1). These microarray data were confirmed by real time RT-PCR analyses of liver specimens from 2-year-old mice that exhibited significant liver fibrosis resulting from the combination of excessive mitochondrial ROS and high fat/calorie intake (Fig 5). The results indicated that the gene expression levels of CC chemokines such as CCL2 (Fig 8A), CCL3 (Fig 8B), CCL4 (Fig 8C), CCL5 (Fig 8D), CCL8 (Fig 8E) and CCL12 (Fig 8F) were significantly increased only by the combination of excessive mitochondrial ROS and high fat/calorie intake, but neither of these two events alone affected CC chemokine production.

**Table 1:** Functional annotation of gene ontology using microarray data.

Mice / Diet	Fold change	Number of changed probes	GO accession number	GO term	Number of elected probes	*p* value
WT / ND vs *Tet-mev-1* / ND	≥ 10	10	No hit	-	-	
	≥ 5	83	No hit	-	-	
WT / HFHSD vs *Tet-mev-1* / HFHSD	≥ 10	16	No hit	-	-	
	≥ 5	70	No hit	-	-	
WT / ND vs WT / HFHSD	≥ 10	69	GO:0006629	lipid metabolic process	11	0.0331
	≥ 5	217	No hit	-	-	
*Tet-mev-1* / ND vs *Tet-mev-1* / HFHSD	≥ 10	53	No hit	-	-	
	≥ 5	208	GO:0005576	extracellular region	34	6.28E-04
			GO:0008009	chemokine activity	6	0.0012
			GO:0006952 GO:0002217 GO:0042829	defense response	16	0.0035
			GO:0042379	chemokine receptor binding	6	0.0035
			GO:0044421	extracellular region part	22	0.0036
			GO:0002376	immune system process	20	0.0263
			GO:0006955	immune response	12	0.0359

WT, wild type; ND, normal diet; HFHSD, high-fat/high-sucrose diet.

### Increased chemokine expression enhanced infiltration of CCR5-positive cells and accelerated liver fibrosis

**Fig 9:**
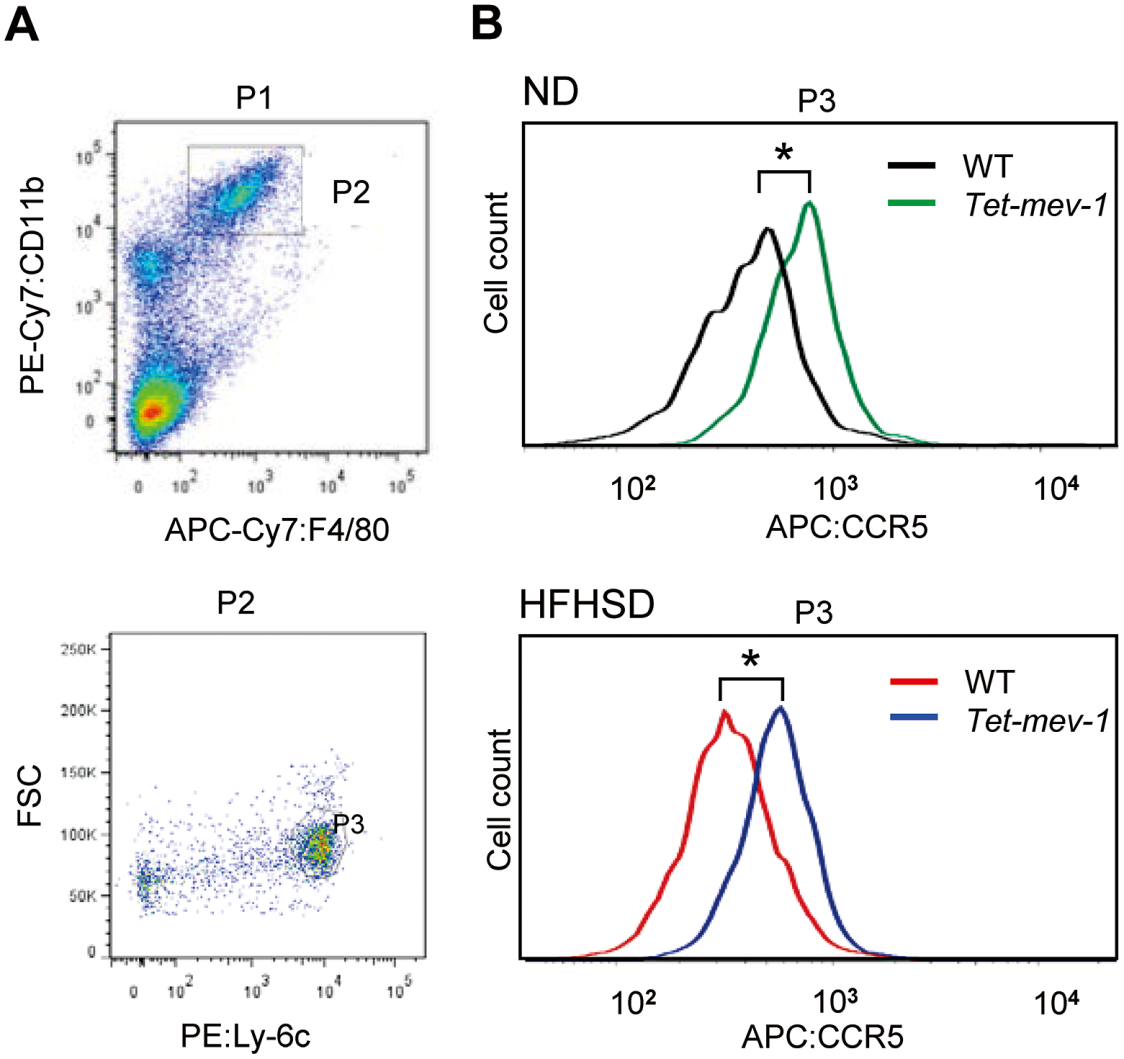
Increased CCR5 expression in peripheral blood monocytes/macrophages of *Tet-mev-1* mice. (**A**) Activated monocytes/macrophages were identified as an F4/80^+^/CD11b^+^/Ly-6C^high^ cell fraction (*P3*) in male wild type (*WT*) and *Tet-mev-1* mice that had been supplied with doxycycline-containing water and were subsequently fed a normal diet (*ND*) or high-fat/high-sucrose diet (*HFHSD*) for 4 months. (**B**) CCR5 expression in these cells was analyzed by measuring the intensities of APC fluorescence bound to anti-CCR5 antibodies using five to six mice per group. The asterisks indicate that the differences between the groups are statistically significant (*p < 0.01).

**Fig 10:**
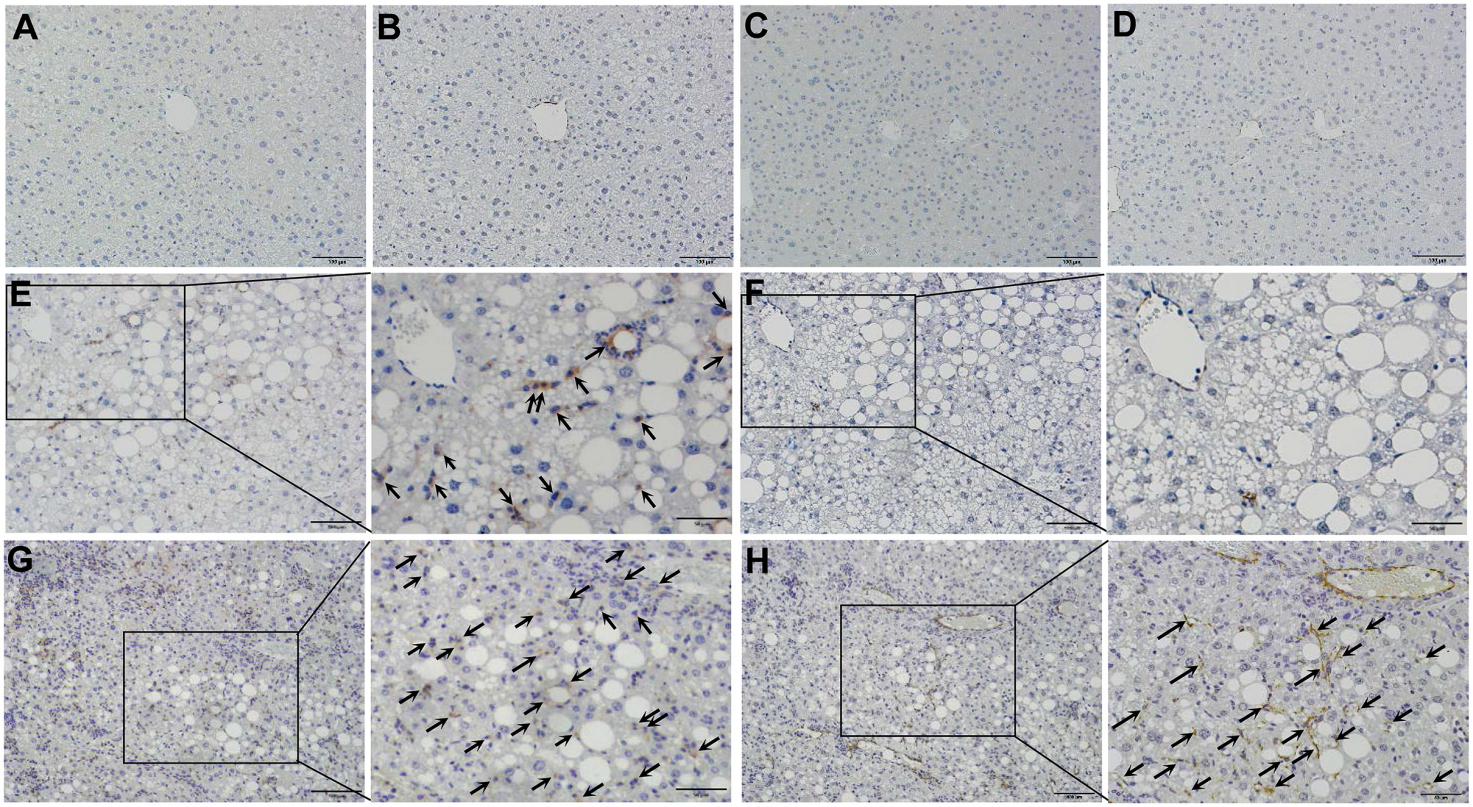
Increased infiltration of CCR5-positive cells and activation of hepatic stellate cells in liver tissues of *Tet-mev-1* mice. Liver specimens were obtained from male wild type (**A**, **B**, **E** and **F**) or *Tet-mev-1* mice (**C**, **D**, **G** and **H**) that had been supplied with doxycycline-containing water and were subsequently fed control, normal chow (**A** to **D**) or a high-fat/high-sucrose diet (**E** to **H**) for 4 months at around the age of 2 years. The tissue sections were subjected to immunohistochemical staining using antibodies against CCR5 (**A**, **C**, **E** and **G**) or α-smooth muscle actin (**B**, **D**, **F** and **H**). CCR5-positive cells are shown by *arrows* in panels **E** and **G**, while α-smooth actin-expressing myofibroblasts were indicated by arrows in panel **H**. Representative images are shown from five mice in each group. *Scale bars*, 100 μm. A part of panels **E** to **H** is presented under high magnification in the corresponding panels on the right.

Based on the above findings showing increased CC chemokine expression, we next examined the expression of CCR5, the major receptor for CCL3 and CCL4, in activated monocytes/macrophages obtained from the peripheral blood of wild type and *Tet-mev-1* mice. The results indicated that the mean CCR5 expression levels were significantly increased in *Tet-mev-1* mice from those in the wild type animals, irrespective of the type of diet provided (Fig 9). Histopathological examination revealed that neither CCR5-positive cells nor αSMA-expressing myofibroblasts were detected in the liver tissues of the wild type (Fig 10A and 10B) or *Tet-mev-1* mice (Fig 10C and 10D) fed a normal diet. Although a significant number of CCR5-expressing non-parenchymal cells were observed in the liver of the wild type mice fed a HFHS diet (Fig 10E), there were no αSMA-positive cells detected in the neighboring regions (Fig 10F). Interestingly, in response to the enhanced CC chemokine expression described above (Fig 8), an increasing number of CCR5-positive mononuclear cells infiltrated into the liver tissues of the *Tet-mev-1* mice fed a HFHS diet (Fig 10G), resulting in the activation of neighboring myofibroblasts stained positive for αSMA (Fig 10H).

## Discussion

In the present study, we examined the direct contribution of mitochondrial oxidative stress to the development of high fat/calorie diet-induced steatohepatitis. We attempted to elucidate the sole effect of oxidative stress under a physiological condition without apparent hepatocellular injury, an effect that can be observed in humans during the aging process. Our results clearly indicated that a combination of mitochondrial oxidative stress and high fat/calorie intake, none of which has any great impact on disease progression by itself, accelerates liver fibrosis by stimulating CC chemokine expression in aged mice. Although our model that includes the aging as one of the important factors for the progression of steatohepatitis is not practical for drug testing, it represents a theoretical model for a plausible paradigm of NASH development.

The *mev-1* was originally identified as an oxidative stress-sensitive short lifespan variant of *C*.*elegans* [19]. The mammalian homolog of the responsible gene is an amino acid substitution mutant (V69E) in mitochondrial SDHC. In a previous study, a transgenic mouse fibroblastic cell line harboring this mutated gene exhibited diminished complex II-III oxidoreductase activity and elevated ROS levels in the mitochondria, resulting in frequent apoptosis and tumorigenesis [20]. Subsequently, *mev-1* transgenic mice were generated in which the mutated V69E gene was ubiquitously overexpressed. Unfortunately, however, this transgenic mouse strain was infertile, precluding its propagation for further studies. Thus, *Tet-mev-1* transgenic mice were generated to control the amount of SDHC V69E protein using a unique Tet-on/off system that equilibrates the transgene expression to the endogenous gene expression levels [13]. Indeed, there was only a modest induction (1.4-fold) of mitochondrial ROS in the *Tet-mev-1* hepatocytes treated with doxycycline (Fig 3A), a results which was comparable to the changes observed during the physiological aging process [21]. It should be noted that a recent study has reported that doxycycline affects mitochondrial structure and functions even at a low concentration [14]. Although parallel experiments using wild type mice of the same age as a control did not show any ROS induction by doxycycline administration, we have to be careful to interpret the data obtained using a mitochondrial toxin.

After confirming the increased ROS production and decreased mitochondrial membrane potential in the *Tet-mev-1* hepatocytes *in vitro*, we conducted a series of *in vivo* experiments to determine whether mitochondrial ROS contribute to the progression of steatohepatitis and, if so, what are the underlying mechanisms responsible for the oxidative stress-induced hepatic fibrogenesis. Two major conclusions have been drawn from the study.

First, the progressive liver fibrosis observed in the *Tet-mev-1* mice fed a HFHS diet was attributed to the increased CC chemokine expression in the liver and the consequent infiltration of CCR5-positive inflammatory cells. This was accompanied by activation of the neighboring hepatic stellate cells stained positive for αSMA. Great attention has been paid to CC chemokines in the pathogenesis of liver fibrosis, and experiments using mice deficient in their receptors (CCR1, CCR2 or CCR5) have clearly shown the direct contribution of CC chemokines to hepatic fibrogenesis induced by carbon tetrachloride administration or bile duct ligation [22, 23]. Similarly, another study has revealed a critical role of CCR2 in the pathogenesis of experimental steatohepatitis induced by a choline-deficient diet [24]. The results of the present study have implicated the role of CC chemokines in the development of steatohepatitis under a more physical condition, which was induced by a small amount of mitochondrial ROS in combination with excess fat/calorie intake.

Second, the combinatorial effect of mitochondrial oxidative stress and excess fat/calorie intake was more apparent in the 2-year-old *Tet-mev-1* mice than in the 1-year-old transgenic animals. This was possibly dependent on the duration and extent of ROS-induced chemokine production in the aged *Tet-mev-1* mice. In addition, an aging cell is generally known to exhibit high levels of oxidative stress [25], and a recent study reported that the Nrf2 signaling, which serves as a master regulator of a highly coordinated antioxidant response, is impaired in aged mice despite the age-related increase in basal oxidative stress, suggesting inadequate protection against oxidative damage in these animals [26].

It should be noted that the effects of high concentrations of free cholesterol (1–1.25% in the diet) on the progression of liver fibrosis have been well documented in experimental steatohepatitis [27, 28]. In addition, dietary cholesterol intake has also been considered important in human NASH development [29]. However, the use of diet containing high concentrations of cholesterol may mask the effects of small amounts of mitochondrial ROS generated in *Tet-mev-1* mice. The HFHS diet used in the present study contained only a small amount (0.035%) of free cholesterol (S1 Table), which by itself did not cause significant fibrosis when given to wild type mice (Figs 4 and 5). This approach is more suitable to examine the combinatorial effects of subclinical mitochondrial ROS and the ordinary fat/calorie intake on hepatic fibrogenesis in humans who do not take an extremely high-cholesterol diet every day. In addition, the results obtained from a comparison between the wild type and *Tet-mev-1* mice that had been given the same doxycycline-containing water and diet excluded the possibility that the altered gut microbiota profile caused by doxycycline administration and/or excess fat/calorie intake accelerated lipopolysaccharide production and subsequent liver fibrosis [30]. Excess fat/calorie intake in *Tet-mev-1* mice presumably increases the preload on the mitochondrial respiratory chain, which leads to a further increase in electron leakage from the deteriorated complex II [4]. Consistent with these results, extensive lipid peroxidation estimated by 4-HNE staining was observed only in the *Tet-mev-1* mice that had been fed a HFHS diet, and not in the same transgenic mice fed normal chow or in the wild type animals fed a HFHS diet (Fig 7). Interestingly, 4-HNE is not only a marker of lipid peroxidation, but also acts as a selective profibrogenic stimulus for activated hepatic stellate cells [31].

The results of the present study also highlighted the important role of genetic factors in the pathogenesis of NASH. Although a “two-hit” theory was originally proposed as the underlying mechanism of NASH development [8], the reason why similar obese patients exhibit different clinical courses (simple steatosis versus NASH) under similar nutritional and environmental conditions remains unknown. In such cases, genetic factors are considered as another critical determinant of NASH development. For example, genetic variants in *PNPLA3* have recently been reported to confer susceptibility to NASH by accelerating fat accumulation in the liver [32]. From this point of view, it is interesting that germline *SDHC* mutations have been reported in several patients [33, 34]. Taken together, these findings may suggest that a certain population has an impaired electron transport system that results in subclinical mitochondrial ROS overproduction, and that excess fat/calorie intake increases the susceptibility to NASH in such individuals [35]. Further studies are needed to explore the genetic predisposition to NASH development in obese patients, which could eventually contribute to earlier diagnosis and intervention for the disease in susceptible or high-risk individuals.

## Supporting Information

S1 Fig Serum biochemical parameters in wild type and *Tet-mev-1* mice fed normal diet. Male wild type (*WT*) and *Tet-mev-1* mice that had been supplied with doxycycline-containing water from the embryonic period were sacrificed at around the age of either 1 (*left*) or 2 years (*right*). The serum levels of alanine aminotransferase (**A**), cholesterol (**B**), and triglyceride (**C**) were determined. The values are expressed as means ± SD from seven to nine mice in each group. *NS*, not significant.
(TIF)

S1 Table Components of a control, normal diet and high-fat/high-sucrose diet used in the study. (DOCX)

S2 Table Relative body weight changes in mice fed a control, normal diet or high-fat/high-sucrose diet. (DOCX)

S3 Table Primer sequences used for quantitative PCR. (DOCX)
