# Supplementary figures and images for: A Combination of Mitochondrial Oxidative Stress and Excess Fat/Calorie Intake Accelerates Steatohepatitis by Enhancing Hepatic CC Chemokine Production in Mice
Source: PLoS One. 2016 Jan 8;11(1):e0146592. doi: 10.1371/journal.pone.0146592 (PMC4706441; doi:10.1371/journal.pone.0146592)

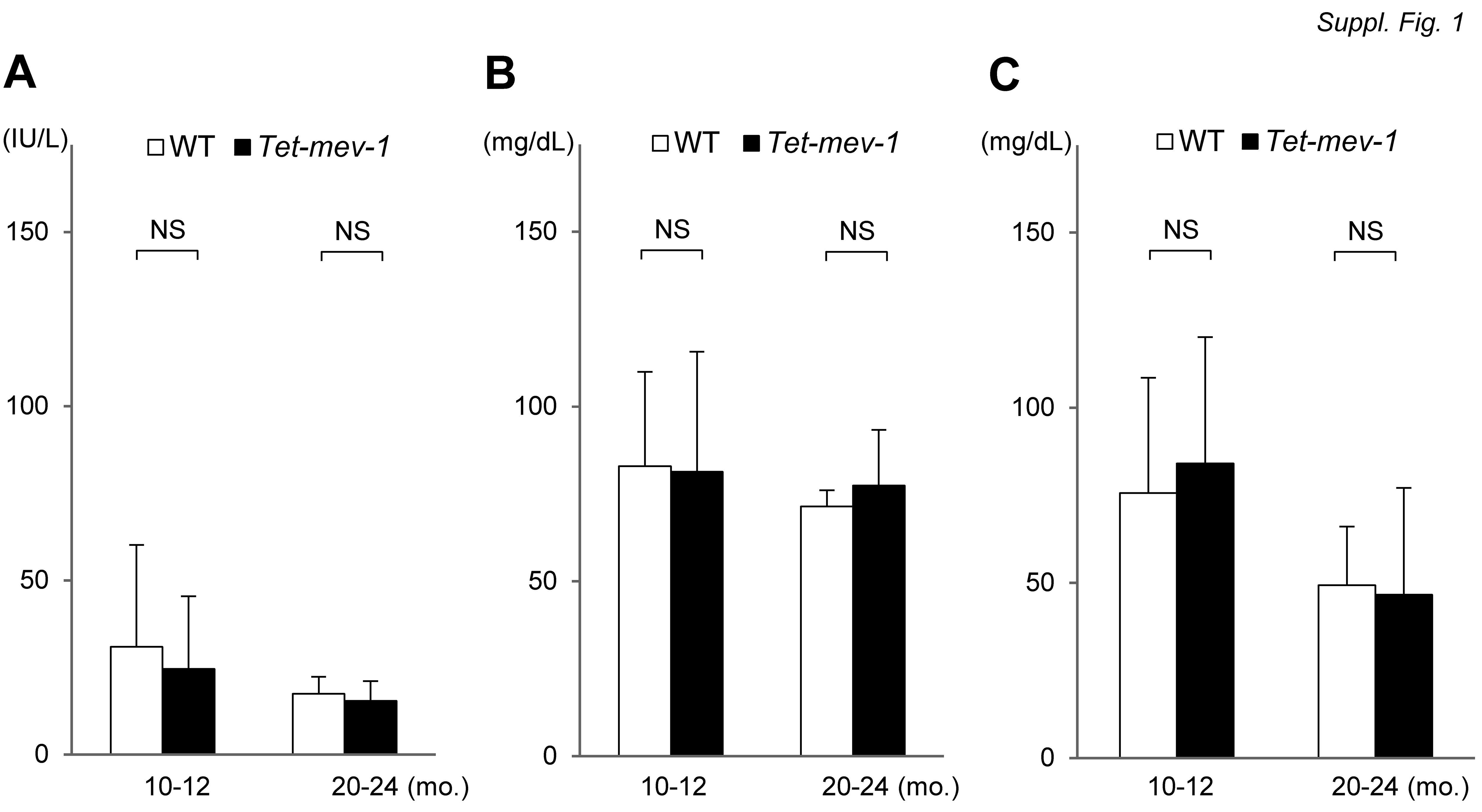

Supplement: S1 Fig — Male wild type (WT) and Tet-mev-1 mice that had been supplied with doxycycline-containing water from the embryonic period were sacrificed at around the age of either 1 (left) or 2 years (right). The serum levels of alanine aminotransferase (A), cholesterol (B), and triglyceride (C) were determined. The values are expressed as means ± SD from seven to nine mice in each group. NS, not significant. (TIF) [file pone.0146592.s001.tif]
