# Supplementary material for: A Combination of Mitochondrial Oxidative Stress and Excess Fat/Calorie Intake Accelerates Steatohepatitis by Enhancing Hepatic CC Chemokine Production in Mice
Source: PLoS One. 2016 Jan 8;11(1):e0146592. doi: 10.1371/journal.pone.0146592 (PMC4706441; doi:10.1371/journal.pone.0146592)
Supplement: S1 Table — (DOCX) [file pone.0146592.s002.docx]

# **Supplementary Table. 1. Components of high-fat/high-sucrose diet and control normal diet used in the study.**

| Components | F2HFHSD | Control |
| --- | --- | --- |
| Water | 8.0 % | 8.9 % |
| Crude protein | 20.7 % | 23.1 % |
| Crude fat | 29.1 % | 5.1 % |
| Cholesterol | 0.035 % | 0.066 % |
| Crude fiber | 5.0 % | 2.8 % |
| Crude ash | 3.2 % | 5.8 % |
| Carbohydrate | 34.0 % | 55.3 % |
| Sucrose | 20.0 % | 0 % |
| Calorie (per 100g) | 481 kcal | 359 kcal |

HFHSD, high-far/high-sucrose diet.
