# Supplementary material for: A Combination of Mitochondrial Oxidative Stress and Excess Fat/Calorie Intake Accelerates Steatohepatitis by Enhancing Hepatic CC Chemokine Production in Mice
Source: PLoS One. 2016 Jan 8;11(1):e0146592. doi: 10.1371/journal.pone.0146592 (PMC4706441; doi:10.1371/journal.pone.0146592)
Supplement: S2 Table — (DOCX) [file pone.0146592.s003.docx]

# **Supplementary Table 2. Relative body weight changes in mice fed control normal diet or high-fat/high-sucrose diet.**

| Age (mo.) | Genotype | Diet | % BW Change (mean ± SD) |
| --- | --- | --- | --- |
| 8-11 | WT | ND | 116.8 ± 12.1 |
|  | WT | HFHSD | 145.0 ± 4.4 * |
|  | *Tet-mev-1* | ND | 111.1 ± 9.8 |
|  | *Tet-mev-1* | HFHSD | 139.8 ± 8.2 * |
| 16-19 | WT | ND | 104.7 ± 1.8 |
|  | WT | HFHSD | 146.0 ± 14.9 * |
|  | *Tet-mev-1* | ND | 113.3 ± 2.4 |
|  | *Tet-mev-1* | HFHSD | 162.4 ± 5.0 * |

WT, wild type; ND, normal diet; HFHSD, high-fat/high-sucrose diet.

*statistically different from data in the same mice fed normal diet (p<0.05).
