# Supplementary material for: A Combination of Mitochondrial Oxidative Stress and Excess Fat/Calorie Intake Accelerates Steatohepatitis by Enhancing Hepatic CC Chemokine Production in Mice
Source: PLoS One. 2016 Jan 8;11(1):e0146592. doi: 10.1371/journal.pone.0146592 (PMC4706441; doi:10.1371/journal.pone.0146592)
Supplement: S3 Table — (DOCX) [file pone.0146592.s004.docx]

# **Supplementary Table 3. Primer sequences used for quantitative PCR.**

| Name | Symbol | Description | Primer sequence |
| --- | --- | --- | --- |
| Ccl2_F | MCP-1 | Monocyte chemotactic protein-1 | 5’-catccacgtgttggctca-3’ |
| Ccl2_R |  |  | 5’-gatcatcttgctggtgaatgagt-3’ |
| Ccl3_F | MIP-1α | Macrophage inflammatory protein-1alpha | 5’-tgcccttgctgttcttctct-3’ |
| Ccl3_R |  |  | 5’-gtggaatcttccggctgtag-3’ |
| Ccl4_F | MIP-1β | Macrophage inflammatory protein-1 beta | 5’-gccctctctctcctcttgct-3’ |
| Ccl4_R |  |  | 5’-ggagggtcagagcccatt-3’ |
| Ccl5_F | RANTES | Regulated on activation, normal T cell expressed and secreted | 5’-tgcagaggactctgagacagc-3’ |
| Ccl5_R |  |  | 5’-gagtggtgtccgagccata-3’ |
| Ccl8_F | MCP-2 | Monocyte chemotactic protein-2 | 5’-ttctttgcctgctgctcata-3’ |
| Ccl8_R |  |  | 5’-gcaggtgactggagccttat-3’ |
| Ccl12_F | MCP-5 | Monocyte chemotactic protein-5 | 5’-ccatcagtcctcaggtattgg-3’ |
| Ccl12_R |  |  | 5’-cttccggacgtgaatcttct-3’ |
| Col1a1_F | Col1a1 | Collagen, type I, alpha 1 | 5’-catgttcagctttgtggacct-3’ |
| Col1a1_R |  |  | 5’-gcagctgacttcagggatgt-3’ |
| Tgfb1_F | Tgfb1 | Transforming growth factor, beta 1 | 5’-tggagcaacatgtggaactc-3’ |
| Tgfb1_R |  |  | 5’-gtcagcagccggttacca-3’ |

F, forward primer; R, reverse primer.
